# Editorial: Beyond the hype: a global perspective on the real-world utility of AI in healthcare research and service delivery

**DOI:** 10.3389/frhs.2026.1957500

**Published:** 2026-08-25

**Authors:** Tonio Schoenfelder, Tom Karl Schaal, Sven Hellbach

**Affiliations:** 1 Department of Health Systems Research, Scientific Institute for Health Economics and Health System Research (WIG2), Leipzig, Germany; 2 Faculty of Health and Healthcare Sciences, University of Applied Sciences Zwickau, Zwickau, Germany; 3 Faculty of Physical Engineering/Computer Sciences, University of Applied Sciences Zwickau, Zwickau, Germany

**Keywords:** AI best-practice, AI regulation and oversight in healthcare, AI-based diagnostics and treatment, bias and fairness in AI in healthcare, clinical decision support systems, large language model (LLM), machine learning in healthcare

When the previous Research Topic in this journal series closed in late 2024, it described digital health services as gaining prevalence worldwide, yet still “perceived as innovations in most healthcare systems, encountering challenges due to the absence of well-established reimbursement channels” (Schoenfelder and Schaal). Germany's Digitale Gesundheitsanwendungen (DiGA) pathway, held up in that editorial as a pioneering route to reimbursement, had by then already grown so demanding in its evidence requirements that “hardly any new DiGA is made available” (Schoenfelder and Schaal, 1).

Artificial intelligence inherits this same tension between promise and proof but sharpens it: where digital health applications mainly had to demonstrate patient benefit, AI additionally raises questions of explainability, workforce trust, and the representativeness of the data it is trained on. A growing “beyond the hype” literature has begun documenting this gap directly: between AI's controlled-trial performance and its real-world impact (2), between diagnostic promise and actual clinical adoption (3), and between regulatory clearance and sustained use in practice (4). It is against this backdrop that the fifteen contributions to this Research Topic, drawn from twelve countries across five continents, examine what AI actually does – and does not yet do – in healthcare research and service delivery.

In this Research Topic, the contributing authors present a variety of perspectives on the real-world application of AI across clinical, administrative, educational, and patient-facing settings. Alblewi surveyed pediatric hematology-oncology physicians in Saudi Arabia and reported that although 74.2% held positive attitudes toward AI, 68.9% had never actually used an AI-based decision-support system – a caution, in the author's words, against confounding anticipated behavior with real experience. Petrica et al. surveyed emergency-department professionals in Romania and identified three distinct attitude clusters toward AI-assisted triage, concluding that the central challenge is cultivating trust and the right cultural environment rather than building smarter algorithms. Yu et al. examined AI readiness among nurses in resource-limited Chinese county hospitals, finding that only 12% had received any AI training and that change fatigue, more than any demographic factor, predicted negative attitudes.

Said et al. surveyed pharmacists in the United Arab Emirates and found confidence in AI's operational benefits but far more caution about its clinical impact, alongside concerns over cybersecurity, data privacy, and job displacement. Giebel et al. surveyed German intensive-care physicians and reported that 80.6% held positive attitudes toward AI, yet more than half struggled to find reliable information about it and fewer than a quarter had used it professionally. Zeng et al. applied psychological network analysis to novice and expert nurses in Chinese maternal and child health settings and identified “AI fear” as the most central node shaping attitudes in both groups. Taken together, these studies consistently demonstrate that positive attitudes toward AI often coexist with limited practical experience, highlighting that successful implementation depends less on technological capability than on trust, education, and organizational readiness.

Beyond attitudes, several contributions focus on the practical conditions required for trustworthy and sustainable AI implementation. Khashu proposed a Decision-Governed Trustworthy AI framework, arguing that trustworthiness is an emergent property of socio-technical systems rather than a fixed attribute of a model, and that existing principle-based guidance remains largely aspirational. Rozikhodjaeva et al. developed and validated a rule-based, explainable clinical decision-support system for diastolic-function assessment during stress echocardiography, matching expert cardiologist diagnosis in 93% of cases while remaining fully auditable – an approach consistent with evidence that explanation quality, more than model complexity, drives clinician trust (5). Bouabida et al. reframed AI as a cognitive partner rather than a replacement for physicians, cataloguing nineteen documented barriers to implementation spanning technical, workflow, and liability domains.

Alkan et al. conducted a systematic review of hospital-efficiency studies using Data Envelopment Analysis and DEA–machine-learning hybrids, finding that healthcare adopts these more sophisticated methods more slowly than other sectors such as finance or energy. Sezgin and Boch proposed a Pediatric AI Implementation Readiness framework after finding that only 19% of FDA-cleared pediatric AI devices had been trained on pediatric data, a gap consistent with a broader shortage of real-world, prospectively validated evidence for clinical AI more generally (6). Collectively, these studies emphasize that explainability, governance, and implementation readiness are emerging as equally important as algorithmic performance itself.

Schaal et al. compared self-assessed and demonstrated digital competence among health students in Germany, Ukraine, and Kazakhstan, extending this research group's earlier work on digital competencies in nursing education and training (7, 8), and found that German students underrated their own skills relative to peers despite producing the most sophisticated literature-search strategies. Hinsche et al. conducted a rapid review of generative AI in nursing education, reporting that ChatGPT passed Taiwan's national nursing licensing examination at 80.75% accuracy while also fabricating nursing diagnoses in a separate case study, and recommended governing the technology rather than banning it outright. A similar picture emerges from nursing care. While digital care applications show promising effects on cognition, mobility, and activities of daily living, the available evidence remains limited by heterogeneous interventions and predominantly low- to moderate-quality studies, underscoring the need for stronger implementation and effectiveness research (9). Together, these educational studies illustrate that preparing future healthcare professionals for AI requires more than technical instruction. Differences in digital competencies and AI literacy call for tailored educational approaches, where AI-supported learning analytics may itself become part of the solution.

The considerable heterogeneity in AI-related digital competencies poses a major challenge for healthcare education. As highlighted by Franke et al. (10), educational strategies should account for different levels of prior knowledge and learning needs. AI-driven learning analytics, as demonstrated by Trommer et al. (11), may help tailor educational pathways and thereby improve learning outcomes.

Binsar et al. analyzed more than 25,000 online mentions of AI-enabled telemedicine and found broadly positive public sentiment, while cautioning that this may partly reflect data-selection effects rather than organic attitudes. MacNeill et al. surveyed the North American public on interest in AI-driven patient-navigation chatbots and found only moderate enthusiasm, concentrated in simple functions such as resource linkage, a pattern consistent with wider evidence that access and digital-literacy barriers can separate stated interest from actual use, particularly in low-resource settings (12).

In conclusion, the fifteen studies of this Research Topic, together with the broader literature cited above, provide a coherent picture of the current state of AI implementation in healthcare, spanning readiness, governance, education, and real-world adoption. As with the digital health applications examined in the previous Research Topic in this series, attitudes toward AI are consistently more positive than actual experience with it, and the barriers to closing that gap are rarely technical: they are questions of training, explainability, governance, and equitable access to representative data. As AI's proliferation continues to reconfigure healthcare infrastructures worldwide, it remains indispensable to close these implementation gaps and extend the benefits of AI-supported care equitably, including to the low-resource and pediatric populations for whom the evidence base remains thinnest. Sustained collaboration among researchers, clinicians, policymakers, and developers, across the same disciplines and countries represented in this volume, will be essential to move the field beyond the hype and toward real-world utility.
